# Immune-related adverse events of anti-PD-1 immune checkpoint inhibitors: a single center experience

**DOI:** 10.3389/fonc.2023.1252215

**Published:** 2023-10-17

**Authors:** Enikő Sebestyén, Nóra Major, Levente Bodoki, Attila Makai, Ingrid Balogh, Gábor Tóth, Zsuzsanna Orosz, Péter Árkosy, Attila Vaskó, Katalin Hodosi, Zoltán Szekanecz, Éva Szekanecz

**Affiliations:** ^1^Department of Oncology, Faculty of Medicine, University of Debrecen, Debrecen, Hungary; ^2^Department of Rheumatology, Faculty of Medicine, University of Debrecen, Debrecen, Hungary; ^3^Department Pulmonology, Faculty of Medicine, University of Debrecen, Debrecen, Hungary; ^4^Department of Laboratory Medicine, Faculty of Medicine, University of Debrecen, Debrecen, Hungary

**Keywords:** immune-checkpoint inhibitors, immune-related adverse events, anti-PD-1, nivolumab, pembrolizumab, Central-Eastern Europe, Hungary

## Abstract

**Objectives:**

Immune checkpoint inhibitors (ICIs) stimulate antitumor immune responses and, in parallel, they might trigger autoimmune and other immunopathological mechanisms eventually leading to immune-related adverse events (irAE). In our study, we assessed patients with malignancies who underwent anti-PD-1 treatment at the University of Debrecen, Clinical Center.

**Patients and methods:**

Between June 2017 and May 2021, 207 patients started ICI treatment at our university. A total of 157 patients received nivolumab and 50 were treated with pembrolizumab. We looked for factors associated with the development of irAEs. In addition to correlation studies, we performed binary logistic regression analysis to determine, which factors were associated with irAEs. We also performed Forward Likelihood Ratio (LR) analysis to determine independent prognostic factors.

**Results:**

At the time of data analysis, the mean duration of treatment was 2.03 ± 0.69 years. ROC analysis determined that 9 or more treatment cycles were associated with a significantly higher risk of irAEs. A total of 125 patients received ≥9 treatment cycles. Three times more patients were treated with nivolumab than pembrolizumab. Of the 207 patients, 66 (32%) developed irAEs. Among the 66 patients who developed irAEs, 36 patients (55%) developed one, 23 (35%) developed two, while 7 (10%) developed three irAEs in the same patient. The most common irAEs were thyroid (33 cases), dermatological (25 cases), pneumonia (14 cases) and gastrointestinal complications (13 cases). Patients who developed irAEs received significantly more treatment cycles (21.8 ± 18.7 versus 15.8 ± 17.4; p=0.002) and were younger at the start of treatment (60.7 ± 10.8 versus 63.4 ± 10.1 years; p=0.042) compared to patients without irAEs. Pembrolizumab-treated patients developed more but less severe irAEs compared to those receiving nivolumab.

**Conclusion:**

ICI treatment is very effective, however, irAEs may develop. These irAEs might be related to the number of treatment cycles and the type of treated malignancy.

## Introduction

Immune checkpoints are cellular proteins that regulate immune responses. When the B7-1/CD80 molecule on antigen-presenting cells (APC) antigen binds to the T-cell CD28 antigen, positive costimulation starts, and the T lymphocytes become activated. On the other hand, if the B7-2/CD86 or the programmed death ligand 1 (PD-L1) molecule on the surface of APC binds to cytotoxic T-lymphocyte antigen 4 (CTLA4) or T-cell PD-1 receptor, respectively, a negative coinhibitory signal is generated, T lymphocyte anergy develops, and antitumor immune responses will be attenuated (1–3). Immune-checkpoint inhibitors (ICI) block CTLA4- or PD-1-mediated coinhibition and thus may restore antitumor immunity (1–4). ICI therapy has become a significant breakthrough in oncology. Numerous CTLA4 (ipilimumab), PD-1 (nivolumab, pembrolizumab) and PD-L1 inhibitors (atezolizumab, durvalumab, cemiplimab, avelumab) have been approved for the treatment of various malignancies (4–8).

Based on the mode of action of ICIs described above, the stimulation of antitumor immune responses may, in parallel, result in the enhancement of autoimmune and other immunological pathways and thus the possible development of immune-related adverse events (irAE) of these drugs (3, 5, 7–12). Such irAEs occur in up to 40% of cases receiving ICI monotherapy (5, 10). While anti-CTLA4 + anti-PD-1 combination therapy result in higher response rates and longer progression-free survival than either agent alone, combination therapy has been associated with more frequent irAEs (up to 95%) (4, 10, 13). Usually irAEs with anti-PD-1 antibodies are less frequent than those with anti-CTLA4 (12).

The irAEs typically start within the first 3 months after the initiation of ICI therapy (5, 10–12, 14). They include endocrine (thyroid, pituitary, diabetes), gastrointestinal (colitis), respiratory (pneumonitis), musculoskeletal (arthritis, manifest autoimmune rheumatic diseases), dermatologic (rash, itching), neurologic (polyneuropathy, aseptic meningitis, demyelination, Guillain-Barré syndrome) and, more rarely, renal (nephritis), hepatobiliary (hepatitis, cholangitis) and ophthalmologic (uveitis, keratitis, retinopathy, dacryoadenitis) manifestations (3, 5, 10–12). These irAEs might have a significant negative impact on the patient’s performance status, which is also a very important factor in treatment planning (5, 10). Among general symptoms, fatigue is the leading complaint with a rate of 16-37% (5, 10). Interestingly, the occurrence and the severity of certain irAEs have been associated with better efficacy of ICI treatment (15).

There have been several recommendations for the monitoring and management of ICI irAEs. IrAEs associated with anti-PD-1 therapeutic agents are generally reversible and well tolerated (5, 16, 17). It is also possible that patients who previously received ICI therapy would develop late-onset irAEs (5, 10, 17). The management of such irAEs highly depends on the grade (G) of severity. In mild cases (Grade 1), except for cardiac and neurologic side effects, only symptomatic treatment (NSAIDs, corticosteroids) is required, and ICI treatment could be continued. In cases of moderate (Grade 2) irAEs, oral corticosteroid treatment is necessary with close monitoring of the symptoms. Grade 3 and 4 irAEs might occur in 20-25% of patients undergoing anti-PD-1 treatment and respiratory, and gastrointestinal irAEs are the most frequent among serious events. In cases of severe (Grade 3) irAEs, ICI therapy needs to be temporarily interrupted along with administering parenteral corticosteroids. ICI therapy may be restarted when the symptoms resolved to Grade 1. Finally, ICI therapy should be terminated permanently in more severe and life-threatening cases (Grade 4), and high-dose parenteral corticosteroids or even synthetic or biologic immunosuppressive drugs can be initiated. The management of these irAEs also require a multidisciplinary approach and consultations with other medical specialties, as well as health professionals and advocacy experts (5, 14, 16–22).

The present study assessed irAEs in patients with malignant solid tumors with anti-PD-1 therapy, either nivolumab or pembrolizumab treatment between 2017 and 2021 at the University of Debrecen. We evaluated the frequency of irAEs, compared these irAEs in nivolumab- versus pembrolizumab-treated patients, and investigated the determinants of irAE development in these patients. To the best of our knowledge, this is the first Hungarian cohort where ICI irAE data were collected and systematically analyzed.

## Patients and methods

### Patients

Between June 2017 and May 2021, ICI treatment was initiated for 207 patients at the Departments of Oncology and Pulmonology, University of Debrecen. Patient characteristics are included in **Table 1**. Among the 207 patients, there were 138 males and 69 females. Their mean age was 64.6 ± 8.2 years and their age at the initiation of ICI therapy was 62.6 ± 9.8 years (**Table 1**). Eventually 157 patients received nivolumab and 50 received pembrolizumab (**Table 1**). At the time of ICI treatment, patients did not receive any additional chemotherapy or radiotherapy. All patients underwent regular follow-ups until the date of data cut, December 31, 2021.

**Table 1:** Clinical characteristics and efficacy results.

	All	Treatment		
		Nivolumab	Pembrolizumab	p value
**Number of patients, n**	207	157	50	
**Female : male ratio**	69:138	50:107	19:31	p=0.422
Age, years^*^	64.6 ± 8.2	64.4 ± 9.9	65.2 ± 11.3	p=0.209
Age at treatment initiation, years^*^	62.6 ± 9.8	62.3 ± 10.1	63.4 ± 11.2	p=0.145
Treatment duration, years^*^	2.03 ± 0.69	2.13 ± 0.90	1.86 ± 0.86	p=0.051
Mean number of cycles, n^*^	16.6 ± 13.7	18.9 ± 19.3	13.9 ± 12.2	p=0.120
**Number of patients with cycles ≥ 9, n (%)**	125 (60)	97 (62)	28 (56)	p=0.466
**Line of treatment, n (%)** ** 1^st^** ** 2^nd^** ** 3^rd^ or more**	29 (14) 159 (77) 19 (9)	4 (2) 138 (88) 15 (10)	25 (50) 21 (42) 4 (8)	***p<0.01***
**Ongoing or past treatment, n (%)** Ongoing Past	55 (27) 152 (73)	40 (25) 117 (75)	15 (30) 35 (70)	p=0.554 p=0.549
**Discontinuation or switch of the first ICI therapy, n (%)** Progression Complete remission Death irAE Patient’s request Unknown All	105 (69) 6 (4) 29 (19) 6 (4) 3 (2) 3 (2) 152 (100)	86 (74) 5 (4) 19 (16) 3 (2) 2 (2) 2 (2) 117 (100)	19 (54) 1 (3) 10 (29) 3 (8) 1 (3) 1 (3) 35 (100)	p=0.078
**PFS** after ICI (months)	16.6 ± 16.0	16.7 ± 16.4	16.1 ± 14.8	p=0.677

^*^Data are expressed as mean ± SD. Significant differences between the nivolumab versus pembrolizumab groups are in **bold italics**. ICI, immune-checkpoint inhibitor; PFS, progression-free survival.

### Data collection and statistical analysis

During data collection, we reviewed the charts of all patients and logged all necessary data into an Excel sheet. Statistical analysis was performed using SPSS version 26 (IBM, Armonk, NY, USA) software. Data are expressed as the mean ± SD for continuous variables and percentages for categorical variables. The distribution of continuous variables was evaluated by the Kolmogorov-Smirnov test. As the distribution of data was not normal, non-parametric tests were used. Continuous variables were compared between groups by the Mann-Whitney test, while nominal variables were compared using the χ^2^ or Fisher’s exact test, as appropriate. Correlations of any two continuous variables were determined by the Spearman’s test. Binary logistic regression analysis was performed to assess prognostic factors for irAEs. Moreover, we analyzed Forward Likelihood Ratio (LR) to determine independent prognostic factors. Receiver Operating Characteristic (ROC) curves show the sensitivity and specificity for every possible cut-offs for a test. P values < 0.05 were considered statistically significant.

## Results

### Descriptive details of ICI therapy

**Figure 1:**
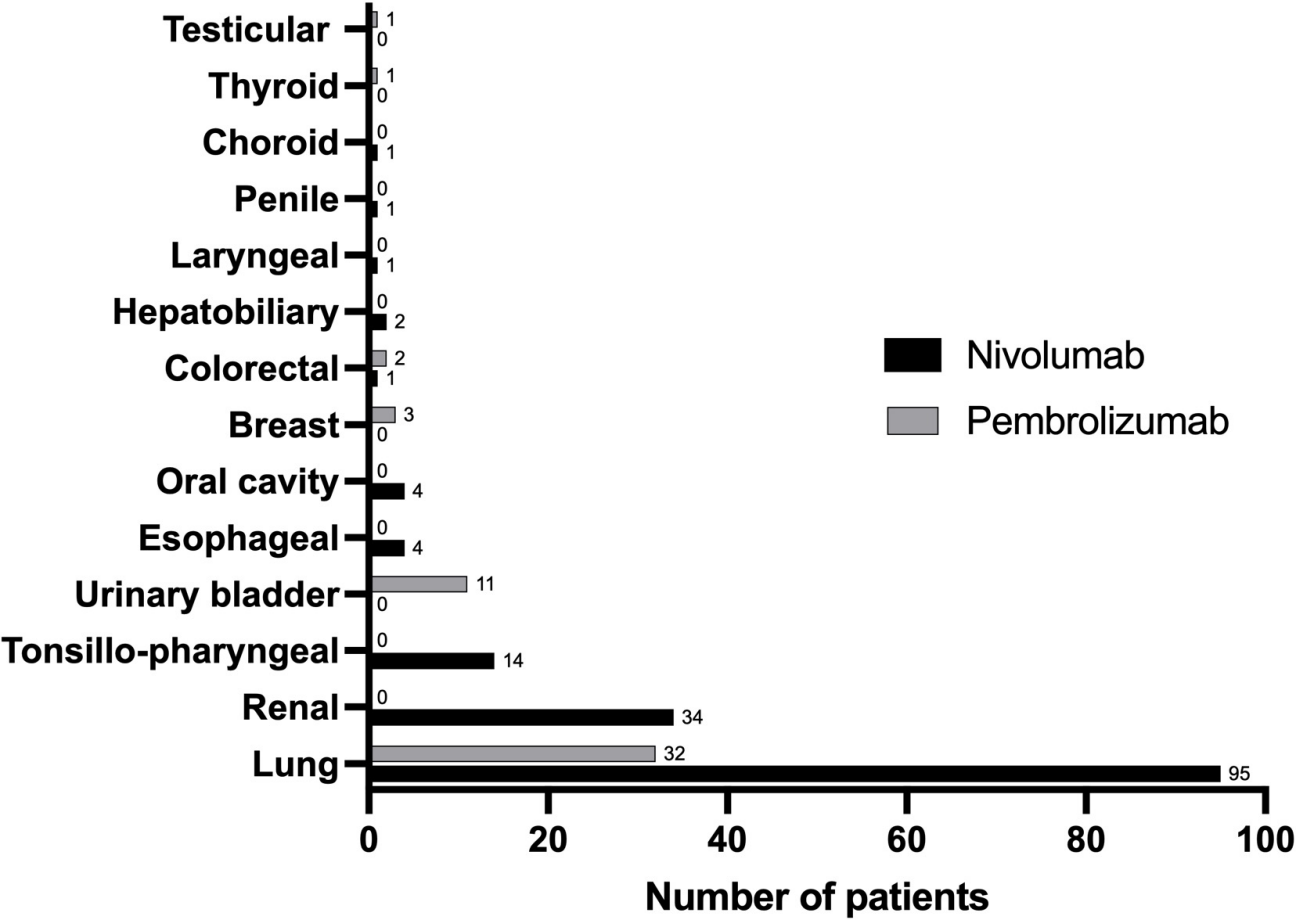
The distribution of indications for nivolumab and pembrolizumab treatment. Numbers show the number of patients with the given type of malignancy.

In our cohort, nivolumab and pembrolizumab were initiated in 157 and 50 patients, respectively (p<0.01; **Table 1**). Among the 207 patients, ICI was started as 1^st^, 2^nd^ or ≥3^rd^ line of treatment in 29, 159 and 19 patients, respectively. At the time of the data cut, the mean treatment duration was 2.03 ± 0.69 years. Altogether 152 patients received anti-PD-1 therapy in the past (73%), while the treatment was still ongoing in 55 patients (27%) (**Table 1**). Among patients who received former anti-PD1 therapy, the reasons for discontinuation or switch were disease progression (105 cases; 69% of patients treated in the past), death (29 cases; 19%), complete remission (6 cases; 4%), irAEs (6 cases; 3%); on patient’s request (3 cases; 2%) or unknown reason (3 cases, 2%) (**Table 1**). Until the data cut, the patients received a mean of 16.6 ± 13.7 cycles of therapy. Altogether 125 patients received 9 treatment cycles or more. The types of malignancies are included in **Figure 1**. The most frequent malignancies were lung (n=127), renal (n=34), tonsillo-pharyngeal (n=14) and urinary bladder cancers (n=11) (**Figure 1**).

In the nivolumab group, the male:female ratio was 107:50. The mean age was 64.4 ± 9.9 years, while that at treatment initiation was 62.3 ± 10.1 years. Among the 157 patients, nivolumab was initiated as 1^st^, 2^nd^ or ≥3^rd^ line of treatment in 4, 138 and 15 patients, respectively. The mean treatment duration was 2.13 ± 0.90 years. Altogether 117 patients (75%) earlier received nivolumab therapy, while this treatment was still ongoing in 40 cases (25%) (**Table 1**). Among patients who received nivolumab therapy in the past, the reasons for discontinuation or switch were disease progression (86 cases; 74% of patients treated in the past with nivolumab), death (19 cases; 16%), complete remission (5 cases; 4%), irAEs (3 cases; 2%), on patient’s request (2 cases; 2%) or unknown reason (2 cases, 2%) (**Table 1**). Our patients received a mean 18.9 ± 19.3 cycles of therapy. Altogether 97 patients received ≥9 treatment cycles (**Table 1**). Among patients receiving nivolumab, the most frequent malignancies were lung (n=95), renal (n=34), tonsillo-pharyngeal (n=14), esophageal (n=4) and oral cavity malignancies (n=4) (**Figure 1**).

In the pembrolizumab group, the male:female ratio was 31:19. The mean age was 65.2 ± 11.3 years, while that at treatment initiation was 63.4 ± 11.2 years. Among the 50 patients, pembrolizumab was initiated as 1^st^, 2^nd^ or ≥3^rd^ line of treatment in 25, 21 and 4 patients, respectively. The mean treatment duration was 1.86 ± 0.86 years. Altogether 35 patients received pembrolizumab treatment in the past (70%), while this therapy was still ongoing in 15 patients (30%) (**Table 1**). Among patients who earlier received pembrolizumab treatment in the past, the reasons for discontinuation or switch were disease progression (19 cases; 54% of patients treated in the past with pembrolizumab), death (10 cases; 29%, complete remission (1 case; 3%), irAEs (3 cases; 8%), on patient’s request (1 case; 3%) or unknown reason (1 case, 3%) (**Table 1**).

Patients received a mean of 13.9 ± 12.2 cycles of therapy. Altogether 28 patients received 9 or more treatment cycles (**Table 1**). Among patients receiving pembrolizumab, the most frequent tumors were lung (n=32) and urinary bladder tumors (n=11) (**Figure 1**).

Considering treatment outcomes, progression-free survival (PFS) rates were calculated in all, as well as nivolumab- and pembrolizumab-treated patients. After anti-PD1 therapy, PFS was observed for 16.6 ± 16.0 months. In the nivolumab- and pembrolizumab-treated subset, PFS durations were 16.7 ± 16.4 and 16.1 ± 14.8, respectively (**Table 1**).

Finally, we analyzed and compared the nivolumab and pembrolizumab groups. There were three times more patients treated with nivolumab than with pembrolizumab. There were also statistically significant differences in the line of treatment as 88% of nivolumab-treated patients received this ICI in 2^nd^ line, while pembrolizumab was used as 1^st^ line treatment in 50% and 2^nd^ line treatment in 42% of the cases (p<0.01). There were no significant differences between the nivolumab- and pembrolizumab-treated patients with respect to genders, age, age at treatment initiation, treatment duration, number of cycles, the number of patients receiving ≥9 cycles, whether anti-PD-1 treatment was in the past or ongoing, the reasons for discontinuation and PFS (**Table 1**). Regarding the types of malignancy, 75% of lung and all 34 renal, 14 tonsillo-pharyngeal, 4 esophageal and 4 oral cavity cancer patients received nivolumab. On the other hand, only 25% of lung, as well as all 11 bladder and 3 breast cancer patients, were treated with pembrolizumab (**Figure 1**).

### Descriptive data on irAEs

**Table 2** includes important information for ICI-related irAEs. Among all 207 patients, 66 (32%) developed altogether 103 irAEs (**Table 2**). Thirty-six patients (55% of patients with irAE) developed one, 23 (35%) developed two, while 7 (10%) developed three different irAEs (**Table 2**). The most frequent irAEs were thyroid (33 cases; 50% of patients with irAE), dermatological (25 cases; 38%), pneumonitis (14 cases; 21%) and gastrointestinal (13 cases; 20%). In addition, nephropathy (7 cases; 11%), hepatopathy (6 cases; 9%), conjunctivitis (2 cases; 3%), pancreatitis (1 case; 1.5%), polyneuropathy (1 case; 1.5%) and polyarthritis (1 case; 1.5%) also occurred (**Table 2**).

**Table 2:** Immune-related adverse events.

	All	Treatment		
		Nivolumab	Pembrolizumab	p value
Number of patients, n	207	157	50	
**Number of patients with irAE, n** **Number of patients with** 1 irAE (%) 2 irAEs (%) 3 irAEs (%) **Total number of irAEs, n**	66 36 23 7 103	45 26 15 4 68	21 10 8 3 35	p=0.078 p=0.566
Number of treatment cycles before the first irAE, n^*^	10.0 ± 10.4	12.0 ± 11.8	7.0 ± 5.7	***p=0.034***
**Severity of irAEs** Grade 1, n (%) Grade 2, n (%) Grade 3, n (%) Mean severity in Grade^*^	62 (60) 36 (35) 5 (5) 1.53 ± 0.63	34 (50) 31 (46) 3 (4) 2.00 ± 0.61	28 (80) 5 (14) 2 (6) 1.35 ± 0.65	***p=0.027***
**irAE subtypes, n (% of patients with irAE)** ** All** Thyroid Skin (rashes) Pneumonitis Gastrointestinal Nephropathy Hepatopathy Conjunctivitis Pancreatitis Polyneuropathy Polyarthritis	**66 (100)** 33 (50) 25 (38) 14 (21) 13 (20) 7 (11) 6 (9) 2 (3) 1 (1.5) 1 (1.5) 1 (1.5)	**45 (100)** 23 (30) 17 (38) 9 (20) 11 (24) 2 (4) 3 (7) 2 (4) - - 1 (2)	**21 (100)** 10 (48) 8 (38) 5 (24) 2 (10) 5 (24) 3 (14) - 1 (5) 1 (5) -	

^*^Data are expressed as mean ± SD. Significant differences between the nivolumab versus pembrolizumab groups are in **bold italics**. Abbreviation: irAE, immune-related adverse event.

Among the 157 nivolumab-treated patients, 45 (29% of nivolumab-treated patients) patients developed 68 irAEs (**Table 2**). In this cohort, 26 patients (58% of nivolumab-treated patients with irAE) developed one, 15 (33%) developed two, while 4 (9%) developed three different irAEs (**Table 2**). In this group, the most frequent IRAEs were thyroid (23 cases; 30% of all nivolumab-treated patients with irAE), dermatological (17 cases; 38%), gastrointestinal (11 cases; 24%) and pneumonitis (9 cases; 20%). We also observed hepatopathy (3 cases; 7%), nephropathy (2 cases; 4%), conjunctivitis (2 cases; 4%) and polyarthritis (1 case; 2%) (**Table 2**).

In the pembrolizumab-treated subgroup, among 50 patients, 21 (42%) developed 35 irAEs (**Table 2**). Here 10 patients (48% of pembrolizumab-treated patients with irAE) developed one, 8 (38%) developed 2, while 3 (14%) developed 3 different irAEs (**Table 2**). In this group, the most frequent irAEs were thyroid (10 cases; 48% of all pembrolizumab-treated patients with irAE), dermatological (8 cases; 38%), nephropathy (5 cases; 24%) and pneumonitis (5 cases; 24%). We also observed hepatopathy (3 cases; 14%), gastrointestinal toxicity (2 cases; 10%), pancreatitis (1 case; 5%) and polyneuropathy (1 case; 5%) (**Table 2**).

IrAEs developed after a mean of 10.0 ± 10.4 cycles in anti-PD-1-treated patients, which occurred after 12.0 ± 11.8 cycles with nivolumab and 7.0 ± 5.7 cycles with pembrolizumab (p=0.034). If more than one irAEs occurred, the time for the first irAE to appear was calculated (**Table 2**).

With respect to irAE severity, the percentage of Grade 1, 2 or 3 irAEs in all anti-PD-1-treated, nivolumab-treated or pembrolizumab treated patients were 60%-35%-5%, 50%-46%-4% and 80%-14%-6%, respectively (**Table 2**). Most irAEs were well-controlled by NSAIDs, corticosteroids or immunosuppressants (data not shown in detail). As discussed above, only six irAEs (3% of all patients) resulted in treatment discontinuation, three in the nivolumab and three in the pembrolizumab group. Treatment discontinuation was needed in one Grade 3, three Grade 2 and two Grade 1 irAE events (**Tables 1** , **2**).

When comparing nivolumab- and pembrolizumab-treated patients, we did not find significant differences in the proportion of patients with irAEs (p=0.078) and in the relative number of different irAEs (p=0.566). When assessing the specific irAEs, nephropathy was significantly more frequent in the pembrolizumab group (p=0.010). Otherwise, there were no differences in the various organ-specific irAEs between the two subgroups. Moreover, irAEs developed after significantly more cycles with nivolumab compared to pembrolizumab (p=0.034). Finally, while nivolumab-associated irAEs were almost equally Grade 1 and 2, pembrolizumab treatment resulted in Grade 1 irAEs in 80% of the cases (p=0.027) (**Table 2**).

### Factors associated with the development of irAEs

When comparing patients with (n=66) and without IRAEs (n=141), patients with irAEs received significantly more treatment cycles (21.8 ± 18.7 versus 15.8 ± 17.4; p=0.002) and were younger at treatment initiation (60.7 ± 10.8 versus 63.4 ± 10.1 years; p=0.042). The number of IRAEs correlated with the number of treatment cycles in a certain patient (R=0.227; p=0.001).

In the simple Spearman’s correlation analysis, the development of irAEs positively and significantly correlated with the length of PFS (R=0.264; p<0.001), the total number of ICI cycles administered (R=0.273; p<0.001) and recent (ongoing) ICI treatment (R=0.183; p=0.008). The number of irAEs also correlated with PFS (R=0.263; p<0.001), the number of ICI cycles (R=0.276; p<0.001) and recent ICI treatment (R=0.193; p=0.005). Finally, the number of ICI cycles administered before the first irAE developed also correlated with PFS (R=0.603; p<0.001) (**Table 3**).

**Table 3:** Results of Spearman’s correlation analysis: significant correlations.

Parameter 1	Parameter 2	R value	p value
Development of irAE	PFS	0.264	<0.001
	Number of ICI cycles	0.273	<0.001
	Ongoing ICI therapy	0.183	0.008
Number of irAEs	PFS	0.263	<0.001
	Number of ICI cycles	0.276	<0.001
	Ongoing ICI therapy	0.193	0.005
Number of ICI cycles before first irAE	PFS	0.603	<0.001

ICI, immune-checkpoint inhibitor; irAE, immune-related adverse event; PFS, progression-free survival.

**Figure 2:**
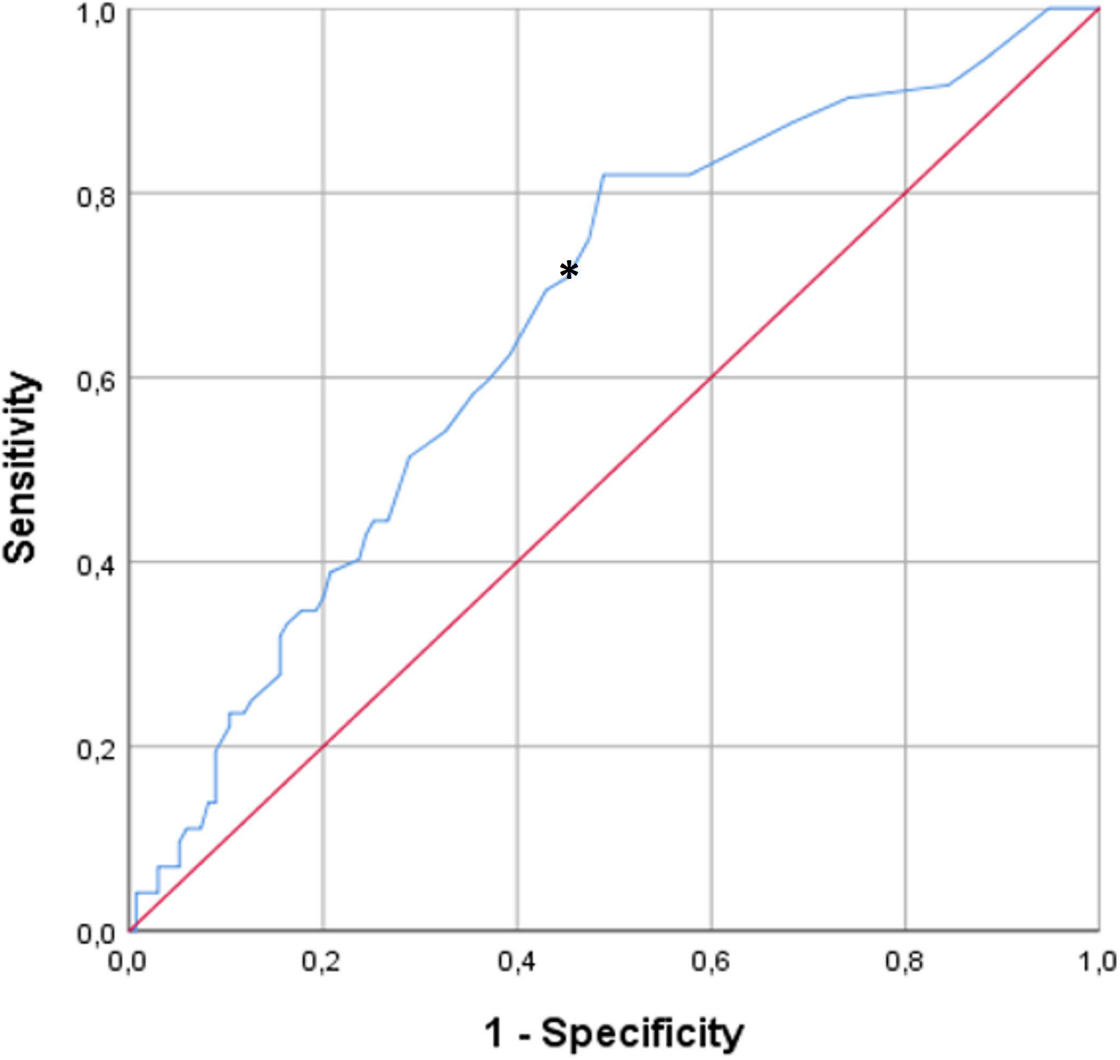
ROC analysis of the association of treatment cycles and the development of irAEs. The asterisk indicates the cut-off of 9 cycles. Nine or more treatment cycles as cut-off resulted in an increased risk for irAEs with an Odds ratio (OR) of 3.328 (95%CI: 1.008-1.042; p=0.004), a sensitivity of 72% and a specificity of 54%.

We performed binary regression analysis to determine possible prognostic factors for the development of irAEs. As defined by the ROC analysis (**Figure 2**), 9 or more treatment cycles as cut-off resulted in an increased risk for irAEs with an Odds ratio (OR) of 3.328 (95%CI: 1.008-1.042; p=0.004), a sensitivity of 72% and a specificity of 54%. The forward LR method also confirmed the same with an OR of 3.578 (95%CI: 1.875-6.831; p<0.001). Nivolumab and pembrolizumab were also compared with respect to the frequency of irAEs. In the binary logistic regression analysis, there was a non-significant tendency showing that pembrolizumab treatment was more frequently associated with irAEs compared to nivolumab (OR: 1.878 [95%CI: 0.980-3.599]; p=0.058). However, in the Forward LR analysis, this difference was statistically significant with an OR of 2.169 (95%CI: 1.089-4.321; p=0.028).

Concerning the specific irAEs, in binary comparisons, patients with thyroid irAEs received more treatment cycles than those without thyroid irAEs (23.0 ± 18.8 versus 16.8 ± 17.7; p=0.04). Patients with pneumonitis also received more treatment cycles (23.1 ± 12.0 versus 17.3 ± 18.3; p=0.022) and had a longer duration of treatment compared to those without pneumonitis (2.5 ± 1.2 versus 2.0 ± 0.9 years; p=0.032). We could not identify any associations between dermatological, gastrointestinal or other specific irAEs or other factors studied.

## Discussion

ICIs have become a significant breakthrough in the treatment of numerous malignancies (4–8). However, due to their mode of action, irAEs may develop during therapy due to the stimulation of anti-cancer immune responses [reviewed in (3, 5, 7, 10–12, 21)]. As there have been few reports in this field in the Central-Eastern European (CEE) region including Hungary, we aimed to share our experience collected on a relatively large cohort of 207 patients treated with PD-1 inhibitors, either nivolumab or pembrolizumab at the Clinical Center of the University of Debrecen.

In our cohort, only 6 patients needed treatment termination due to irAEs. Eventually one-third of the patients developed at least one irAE, after a mean 10 treatment cycles. IrAEs can occur early, while late-onset irAEs are difficult to predict with available tools and, consequently, are hard to prevent (12). Half of these patients had only one irAE, while one-third of them had two and only 10% had three. In accordance with the literature (3, 5, 7, 10–12, 21), the most frequent irAEs were thyroid, skin, diseases, pneumonitis and gastrointestinal conditions. We did not observe any myocarditis (23) or neurotoxicity (24) except for one case of polyneuropathy. In general, 60% of the patients developed Grade 1 irAEs. Most irAEs could be well-controlled using internationally accepted oncology and rheumatology protocols (5, 14, 16, 18–21) and national recommendations (5) and did not require treatment discontinuation. Indeed, irAEs with anti-PD-1 are less frequent than those with anti-CTLA4 (12) and in clinical trials the discontinuation rates are 3-8% (12).

When comparing the two anti-PD-1 agents, in our study, pembrolizumab was twice more often associated with irAEs compared to nivolumab. On the other hand, regarding severity, nivolumab treatment was associated with relatively less Grade 1 but more Grade 2 irAEs compared to pembrolizumab suggesting that pembrolizumab treatment results in milder irAEs. In most systematic reviews, meta-analyses, and comparative assessments, nivolumab and pembrolizumab had similar safety and tolerability profiles (25–28). Therefore, the differences found in our study suggesting that pembrolizumab might cause irAEs more often but these irAEs are milder might be due to other conditions. For example, three times more patients were treated with nivolumab compared to pembrolizumab in this cohort. In our study, pembrolizumab was used earlier, more often in 1^st^ line. Moreover, there were major differences in treatment indications. For example, pembrolizumab was administered to mostly patients with lung cancer. It has not been established, how the underlying malignancy type influences irAE development, severity, and outcomes (3, 5, 7, 10, 11, 21). Thus, it is difficult to directly compare these two ICIs due to heterogeneity in the treatment environment.

Our results confirmed those from others, suggesting that irAEs might show associations with ICI efficacy (12, 15, 29, 30). Moreover, pneumonitis has been suggested to be predictive of favorable outcomes in patients receiving anti-PD-1 antibodies (12, 31). In other studies, risk factors include pre-existing autoimmune diseases, especially those that are active at the time of ICI initiation. In addition, treatment-related factors, such as the type of ICI (anti-PD-1 versus anti-CTLA4), combination of ICIs, as well as intrinsic factors including tumor and genetic heterogeneities, cancer type, tumour microenvironment and the microbiota might also influence the development of autoimmune irAEs (12, 32, 33).

There have been numerous recommendations for the management and possible prevention of autoimmune irAEs (5, 14, 16, 18–21, 34). In our cohort, 60% of irAEs were Grade 1 and most irAEs were easy-to-control and only very few patients required the discontinuation of ICI therapy. As discussed above, many irAEs occur relatively early, in our case, after a mean of 10 treatment cycles. Several preventive strategies and pretreatment assessments of target organ function have been implemented in preventing chemotherapy-related toxicities, which are more predictable than irAEs. With respect to irAEs, no evidence-based algorithms for active surveillance of such events have become available. Most proposed strategies have been based on expert opinion (5, 12, 16, 20). Very few of our patients required cessation of ICI therapy. In most cases, rechallenge after ICI discontinuation is safe and do not lead to repeated irAEs (35).

The strength of our study is that it might be the largest CEE cohort with respect to irAEs associated with ICI therapy. Moreover, we could include a relatively high number of patients from one center and perform multiple analyses to understand the determinants of irAEs. Of course, this study might also have limitations including its single center nature and the solely clinical approach to these issues.

## Conclusions

In our cohort of 207 patients treated with nivolumab or pembrolizumab, we achieved a 16-month PFS with both anti-PD-1 agents. One-third of patients developed irAEs, mostly in Grade 1 and did not require treatment discontinuation in all but 6 cases. There were no major differences between the two drugs in general. However, pembrolizumab seemed to be associated with irAEs more frequently, but these irAEs were less severe compared to those of nivolumab, which could be explained by differences in indications, patient numbers, and other factors. Finally, our results also suggest a close relationship between ICI efficacy as determined by PFS and irAEs. Despite the possible limitations of our study, we collected and analyzed data in the CEE region and provided more information on ICI-related irAEs for practicing physicians (34).

## Data availability statement

The raw data supporting the conclusions of this article will be made available by the authors, without undue reservation.

## Ethics statement

The studies involving humans were approved by University of Debrecen Central Ethics Committee. The studies were conducted in accordance with the local legislation and institutional requirements. Written informed consent for participation was not required from the participants or the participants’ legal guardians/next of kin because We only used patient data but patients could not be identified so we did not need consent.

## Author contributions

ES, ÉS, ZS: conceptualization, drafting and finalization of manuscript. NM, LB, AM, IB, ZO, PÁ, AV: patient recruitment, patient examination, obtaining data GT: laboratory assessments, data provider KH: statistical analysis, data curation All authors work on behalf of the Hungarian OncoRheumatology Network (HORN) initiative. All authors contributed to the article and approved the submitted version.
